# Correction: Gas sensing performance at room temperature of nanogap interdigitated electrodes for detection of acetone at low concentration

**DOI:** 10.1039/c8ra90007h

**Published:** 2018-02-07

**Authors:** Q. Nguyen Minh, H. D. Tong, J. Baggerman, A. Kuijk, S. P. Pujari, J. F. van der Bent, P. Beekman, C. J. M. van Rijn

**Affiliations:** Laboratory of Organic Chemistry, Wageningen University and Research Stippeneng 4, 6708 WE Wageningen The Netherlands cees.vanrijn@wur.nl +31-317-482370; Nanosens IJsselkade 7 7201 HB Zutphen The Netherlands; Ceramics and Biomaterials Research Group, Ton Duc Thang University Ho Chi Minh City Vietnam tongduyhien@tdt.edu.vn; Faculty of Applied Sciences, Ton Duc Thang University Ho Chi Minh City Vietnam; HU University of Applied Sciences Utrecht Oudenoord 700, 3513 EX Utrecht The Netherlands; Medical Cell Biophysics Group, MIRA Institute for Biomedical Engineering and Technical Medicine, Faculty of Science and Technology, University of Twente 7522 NB Enschede The Netherlands

## Abstract

Correction for ‘Gas sensing performance at room temperature of nanogap interdigitated electrodes for detection of acetone at low concentration’ by Q. Nguyen Minh *et al.*, *RSC Adv.*, 2017, **7**, 50279–50286.

The authors regret that in the original article the authorship was incorrect. J. Baggerman and S. P. Pujari were not included in the original author list. Additionally, J. F. van der Bent was spelled incorrectly in the original article. The correct author list is as presented above.

The Royal Society of Chemistry apologises for these errors and any consequent inconvenience to authors and readers.

## Supplementary Material

